# Redesigning Protein Cavities as a Strategy for Increasing Affinity in Protein-Protein Interaction: Interferon-***γ*** Receptor 1 as a Model

**DOI:** 10.1155/2015/716945

**Published:** 2015-04-28

**Authors:** Jiří Černý, Lada Biedermannová, Pavel Mikulecký, Jiří Zahradník, Tatsiana Charnavets, Peter Šebo, Bohdan Schneider

**Affiliations:** Laboratory of Biomolecular Recognition, Institute of Biotechnology, Academy of Sciences of the Czech Republic, Vídeňská 1083, 142 20 Prague, Czech Republic

## Abstract

Combining computational and experimental tools, we present a new strategy for designing high affinity variants of a binding protein. The affinity is increased by mutating residues not at the interface, but at positions lining internal cavities of one of the interacting molecules. Filling the cavities lowers flexibility of the binding protein, possibly reducing entropic penalty of binding. The approach was tested using the interferon-*γ* receptor 1 (IFN*γ*R1) complex with IFN*γ* as a model. Mutations were selected from 52 amino acid positions lining the IFN*γ*R1 internal cavities by using a protocol based on FoldX prediction of free energy changes. The final four mutations filling the IFN*γ*R1 cavities and potentially improving the affinity to IFN*γ* were expressed, purified, and refolded, and their affinity towards IFN*γ* was measured by SPR. While individual cavity mutations yielded receptor constructs exhibiting only slight increase of affinity compared to WT, combinations of these mutations with previously characterized variant N96W led to a significant sevenfold increase. The affinity increase in the high affinity receptor variant N96W+V35L is linked to the restriction of its molecular fluctuations in the unbound state. The results demonstrate that mutating cavity residues is a viable strategy for designing protein variants with increased affinity.

## 1. Introduction

In studying specificity and affinity of protein-protein interactions, the main focus is traditionally on the structural properties of the interface, for example, complementarity of the residue composition, hydrogen-bonding networks, and the role of hydration [1]. However, there is also a significant contribution of the conformational dynamics to the binding affinity. Analysis of molecular dynamics simulations of 17 protein-protein complexes and their unbound components with quasi-harmonic analysis [2] concluded that the protein flexibility has an important influence on the thermodynamics of binding. Moreover, changes in the protein conformational dynamics may lead to substantial changes in affinity to binding partners without an apparent structural change of the complex. For example, reorganization of the hydrogen bonding networks and solvent bridges of the interacting molecules upon mutation, which was accompanied only by subtle structural changes, leads to radically different binding free energy [3, 4]. A recent work [5] shows that the apparent change in the amino acid dynamics determined by NMR spectroscopy is linearly related to the change in the overall binding entropy and also that changes in side-chain dynamics determined from NMR data can be used as a quantitative estimate of changes in conformational entropy [6, 7]. Also, an analysis of crystallographic B-factors has revealed a significant decrease of flexibility of residues exposed to solvent compared to flexibility of residues interacting with another biomolecule and further compared to their flexibility in the protein core [8]. This “freezing” of atoms upon complexation and in the protein core is only slightly larger for the side chain atoms than for the main chain atoms. Entropic cost specific for side-chain freezing has been computationally evaluated as a small, but important contribution to the thermodynamics of binding [9, 10]. These results indicate that changes in amino acid conformational entropy upon binding contribute significantly to the free energy of protein-protein association.

However important the interaction interface is for the affinity, the interaction is influenced by the whole composition of the cognate molecules, so that modulation of affinity can be achieved by changing other residues than residues at the interface. One such possible alternative approach would be filling cavities in one of the binding partners, thus influencing the stability and dynamics of the interacting proteins [11–14]. Thermodynamic consequences of introducing cavity-filling mutations have been discussed for residues at the interaction interface [15–17] showing that filling the interfacial cavity increases affinity due to both gain in binding enthalpy and a loss in binding entropy, the latter being attributed to a loss of conformational degrees of freedom. It has been shown that interactions between the internal “core” residues is responsible for the folding and thermal stability of a protein [18]. Here, we decided to test whether the protein-protein affinity could be increased by mutations not on the interface, but in cavities inside one of the cognate protein molecules.

This study follows our previous article [21] in which we designed mutations increasing the affinity of human interferon-*γ* receptor 1 (IFN*γ*R1) towards its natural cognate molecule interferon-*γ* (IFN*γ*), an important protein of innate immunity [22, 23]. Here, we retain this model system and the main contours of the protocol but replace the search for interface mutations by searching for mutations in the receptor cavities in order to further increase its interaction affinity to IFN*γ* and our computer analysis revealed four such cavity mutants. Combining one of these cavity mutations with the best variant designed in our previous study led to a sevenfold increase in affinity compared to the wild-type receptor. We show that the affinity increase in this mutant is related to the restricted flexibility of amino acids in the unbound state of IFN*γ*R1.

## 2. Materials and Methods

### 2.1. Outline of the Protocol

Our computational predictions are based on the analysis of crystal structures of complexes between IFN*γ* and the extracellular part of IFN*γ*R1, namely, the structures of PDB codes 1fg9 [19] and 1fyh [20] that contain four crystallographically independent IFN*γ*/IFN*γ*R1 complexes. Throughout the paper, IFN*γ*R1 residues are numbered as in UniProt entry P15260. We used the empirical force field implemented in the software FoldX [24] to search for mutations within the positions lining the internal cavities of IFN*γ*R1 molecule that would increase its stability and/or its affinity to IFN*γ*. All designed mutants of IFN*γ*R1 were subsequently expressed and purified and their affinity to a “single-chain” form of IFN*γ* (IFN*γ*SC, [25]) was measured. Individual steps of the computational protocol as well as experimental procedures are described below.

### 2.2. *In Silico* Design of Variants

The program 3V [26] was used to identify internal cavities in all four available structures of IFN*γ*R1 molecules complexed with IFN*γ*. In total, 52 cavity-lining residues, which were identified as encapsulating the cavities in at least one of the four structures, were extracted using the VMD program [27]. Each of 52 amino acid residues identified as lining the internal receptor cavities was mutated in all four crystal IFN*γ*/IFN*γ*R1 complexes to 20 amino acid residues using the “positionscan” and “analyzecomplex” FoldX keywords. This represented 52 × 4 × 20 mutations (including self-mutations leading to ΔΔ*G* = 0). Three types of changes of free energy (ΔΔ*G*) were calculated using the program FoldX:

1. “ΔΔ*G* of folding of IFN*γ*R1 in complex” gauged the influence of mutations on the stability of the whole IFN*γ*/IFN*γ*R1 complex;
2. “ΔΔ*G* of folding of free IFN*γ*R1” estimated the effect of mutations on the stability of the isolated receptor;
3. “ΔΔ*G* of binding” of complex between IFN*γ*R1 and IFN*γ* estimated the change of the interaction between the receptor molecule and the rest of the complex.

### 2.3. Modeling

IFN*γ*R1 models are based on PDB structures 1fg9 [19] and 1fyh [20]. Missing residues in both structures were added using Modeller suite of programs [28]. The lowest energy loop models were used for further calculations.

### 2.4. Molecular Dynamics (MD) Simulations

MD simulations were run using GROMACS suite of programs to test the stability and dynamic properties, including analysis of values of root means square fluctuations (RMSF) [29] and the effect of variable geometry on prediction of changes of interaction free energy (ΔΔGs), of the IFN*γ*/IFN*γ*R1 complexes (PDB codes 1fyh and 1fg9). More detailed protocol of MD and FoldX calculations follows.

### 2.5. Protocol of Molecular Dynamics (MD) Calculations

For the MD simulations the following setup was used: protonation state was determined by pdb2gmx program using parameters provided by the OpenMM [30] Zephyr [31] program. Implicit solvation (GBSA, *ε* = 78.3, with collision interval of 10.99 fs) was used in combination with parm96 force field [32]. OpenMM Zephyr implementation of GPU accelerated version of GROMACS [29] suite of programs was used to simulate the systems. The initial crystal structures were optimized and the simulation was propagated at 300 K with the time step of 2 fs. RMSF (root-mean square fluctuations) of atoms in the analyzed proteins were calculated from the 100 ns trajectory to estimate flexibility of residues; they were calculated by g_rmsf program in 5 ns windows.

### 2.6. Construction, Expression, and Purification of Recombinant IFN*γ*R1 Variants

We followed the protocols from our previous study [21] for all proteins produced in this study. All selected IFN*γ*R1 variants were prepared, expressed, and successfully purified to homogeneity by the following protocol.

Codon-optimized synthetic gene (GenScript) encoding extracellular domain of human IFNgR1 (residues 18–245) was cloned into the pET-28b(+) vector (Novagen) using* NcoI* and* XhoI* restriction enzymes in frame with N-terminal start codon and C-terminal HisTag. The QuikChange II Site-Directed Mutagenesis Kit (Agilent Technologies) was used for mutating the IFN*γ*R1 gene according to manufacturer's manual using primers listed below. Primers were designed by web-based PrimerX program (http://www.bioinformatics.org/primerx/).

**Figure 1:**
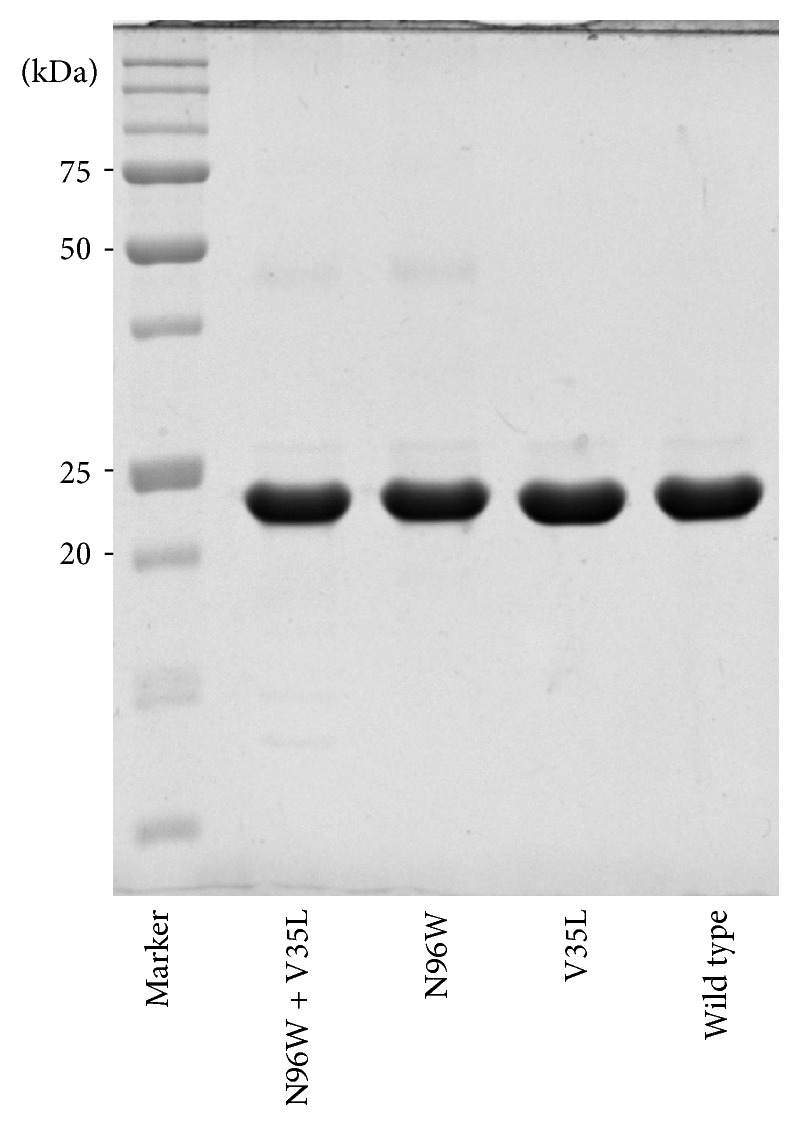
Nonreducing 12.5% SDS-PAGE gel of selected monomeric refolded recombinant His-tagged IFN*γ*R1 variants. Proteins were extracted from inclusion bodies by 8 M urea, further purified on Ni-NTA agarose, and dialyzed, and monomeric fraction was separated on gel filtration column (see above). IFN*γ*R1 with C-terminal His-Tag migrates at a molecular mass of 23 kDa when analyzed on nonreducing SDS-PAGE gel.

The recombinant IFN*γ*R1 variants were expressed in* Escherichia coli* BL21 (*λ*DE3) in LB medium containing 60 *μ*g/mL of kanamycin at 37°C for 4 hours after induction by 1 mM IPTG. Harvested cells by centrifugation (8,000 g, 10 min, 4°C) were disrupted by ultrasound in 50 mM Tris buffer pH 8 and centrifuged at 40,000 g, 30 min, 4°C, and inclusion bodies were dissolved in 50 mM Tris buffer pH 8 containing 8 M urea and 300 mM NaCl to extract protein that was further affinity-purified on Ni-NTA agarose (Qiagen) in the same buffer. Protein was eluted from resin by 250 mM Imidazole pH 8 in previous buffer and refolded by dialysis against 100 mM Tris-HCl pH 8, 150 mM NaCl, 2.5 mM EDTA, 0.5 mM Cystamine, and 2.5 mM Cysteamine overnight at 4°C. Final purification of monomeric receptor variants was performed at 4°C on a HiLoad 16/600 Superdex 200 pg (GE Healthcare) equilibrated by PBS buffer pH 7.4 (Figure 1). Monodispersity of the purified receptor protein was verified by dynamic light scattering (DLS) using Malvern Zetasizer Nano ZS90 instrument (data not shown).

### 2.7. Primers

Mutagenesis primers are designed for the introduction of single residue substitution into IFN*γ*R1 WT. Mutated nucleotides are underlined. We have the following:

 
V35L
 
Forward: 5′-GTCCCGACCCCGACCAACTTGACGATTGAAAGTTACAAC-3′
 
Reverse: 5′-GTTGTAACTTTCAATCGTCAAGTTGGTCGGGGTCGGGAC-3′
 
A114E

 
Forward: 5′-GAAAGAATCAGCGTATGAAAAATCGGAAGAATTCGCC-3′
 
Reverse: 5′-GGCGAATTCTTCCGATTTTTCATACGCTGATTCTTTC-3′
 
D124N
 
Forward: 5′-CGCCGTGTGCCGTAATGGCAAAATCG-3′
 
Reverse: 5′-CGATTTTGCCATTACGGCACACGGCG-3′
 
H222Y
 
Forward: 5′-CTGAAGGCGTTCTGTATGTCTGGGGTGTC-3′
 
Reverse: 5′-GACACCCCAGACATACAGAACGCCTTCAG-3′

### 2.8. Construction, Expression, and Purification of IFN*γ*SC

Recombinant interferon gamma in so-called single chain form (IFN*γ*SC) described by [25] was cloned into pET-26b(+) vector (Novagen) using* NdeI* and* XhoI* restriction enzymes in frame with N-terminal start codon not to have no peptide leader nor tag.

The recombinant IFN*γ*SC was expressed in* E. coli* BL21 (*λ*DE3) in LB medium containing 60 *μ*g/mL of kanamycin at 30°C for 4 hours after induction by 1 mM IPTG. Harvested cells by centrifugation (8,000 g, 10 min, 4°C) were disrupted by ultrasound in 20 mM Na-Phosphate buffer pH 7.3 and centrifuged at 40,000 g, 30 min, 4°C, and soluble fraction was further purified on SP Sepharose HP (GE Healthcare) using linear gradient of NaCl and further purified to homogeneity by gel filtration in same procedure as IFN*γ*R1 receptor (see above).

### 2.9. Biophysical Characterization of the Studied Proteins

Melting temperatures of the receptor variants were measured using fluorescence-based thermal shift assay and for selected mutants by CD melting experiments. Interactions between IFN*γ*R1 variants and IFN*γ*SC were measured by the technique of surface plasmon resonance (SPR) as discussed in our previous study [21]. Experimental procedures are detailed below.

### 2.10. CD Measurements

CD spectra were recorded using “Chirascan-plus” (Applied Photophysics) spectrometer in steps of 1 nm over the wavelength range of 190–260 nm. Samples at a concentration of 0.2 mg/mL were placed into 0.05 cm path-length quartz cell to the thermostated holder and individual spectra were recorded at the temperature of 25°C. The CD signal was expressed as the difference between the molar absorption of the right- and left-handed circularly polarized light and the resulting spectra were buffer subtracted. To analyze the ratio of the secondary structures we used the CDNN program provided with Chirascan CD spectrometer [33]. For CD melting measurements, samples at a concentration of 1.5 mg/mL were placed into 10 mm path-length quartz cell to the thermostated holder and CD signal at 280 nm was recorded at 1°C increment at rate of 1.0°C/min over the temperature range of 25 to 65°C with an averaging time of 10 seconds. CD melting curves were normalized to relative values between 1.0 and 0.0.

### 2.11. Thermostability of the IFN*γ*R1 Variants by Thermal-Based Shift Assay

Melting temperature (*T*_*m*_) curves of the WT and selected variants were obtained from fluorescence-based thermal shift assay (TSA) using fluoroprobe. Experiment was performed in “CFX96 Touch Real-Time PCR Detection System” (Bio-Rad) using FRET Scan Mode. The concentration of fluorescent SYPRO Orange dye (Sigma Aldrich) was 8-fold dilution from 5000-fold stock and protein concentration was 2 *μ*L in final volume of 25 *μ*L. As a reference we used only buffer (PBS buffer pH 7.4) without protein. Thermal denaturation of proteins was performed in capped “Low Tube Strips, CLR” (Bio-Rad) and possible air bubbles in samples were removed by centrifugation immediately before the assay. The samples were heated from 20°C to 75°C with stepwise increment of 0.5°C per minute and a 30 s hold step for every point, followed by the fluorescence reading. Data subtraction by reference sample was normalized and used for first derivative calculation to estimate the melting temperature.

### 2.12. SPR Measurements

His-tagged receptor molecules were diluted to concentration of 10 *μ*g/mL in PBST running buffer (PBS pH 7.4, 0.005% Tween 20) and immobilized on a HTG sensor chip activated with Ni^2+^ cations at a flow rate 30 *μ*L/min for 60 s to gain similar surface protein density. Purified IFN*γ*SC was diluted in running buffer to concentrations ranging from 0.1 to 9 nM and passed over the sensor chip for 90 seconds at a flow rate 100 *μ*L/min (association phase). Dissociation was measured in the running buffer for 10 min at the same flow rate. Correction for nonspecific binding of IFN*γ*SC to the chip surface was done by subtraction of the response measured on uncoated interspots and reference channel coated with His-tagged Fe-regulated protein D (FrpD) from Neisseria meningitides [34]. Data were processed in the ProteOn Manager software (version 3.1.0.6) and the doubly referenced data were fitted to the 1 : 1 “Langmuir with drift” binding model.

## 3. Results and Discussion

### 3.1. Internal Cavities Identified in IFN*γ*R1

**Figure 2:**
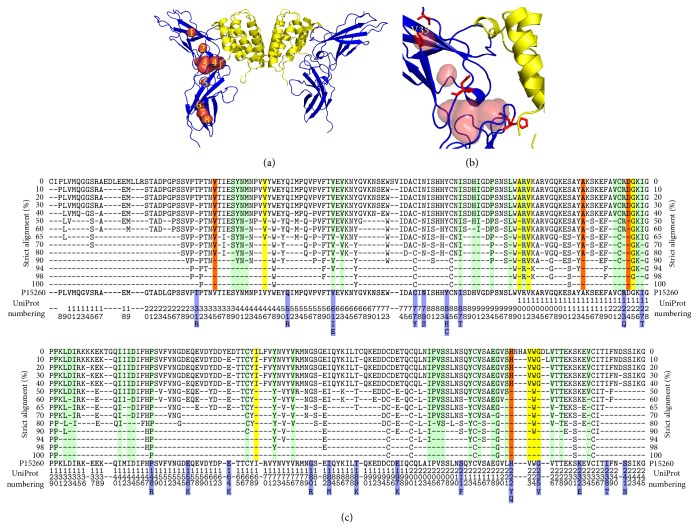
(a) The complex between IFN*γ* and the extracellular part of its receptor 1 (IFN*γ*R1) from crystal structure of PDB code 1fg9 [19]. The two IFN*γ*R1 molecules are drawn as blue cartoon and IFN*γ* homodimer as yellow cartoon. The eight identified cavities in the receptor molecule are shown as numbered red surfaces. (b) A close-up of the mutated cavities. The receptor cavities are drawn as red surface and residues selected for mutations as red sticks; valine 35 is labeled. (c) Residue conservancy calculated by strict alignment of 32 sequences of the extracellular part of IFN*γ*R1 from 19 species. The residues lining the cavities and not suitable for mutation are highlighted in green, those selected by FoldX as mutable in yellow, and the residues selected for mutations after MD simulations are in red (they are also listed in Table 1). Blue highlights show IFN*γ*R1 mutants occurring naturally in humans. Percentages of the conservation are shown on the left and right sides; analyzed sequence (residues 6–245 of the UniProt entry P15260) is shown at the bottom of the alignment.

The cavity analysis revealed generally different number and size of cavities for each IFN*γ*R1 crystal structure; their characteristics are listed in Table 1; their location in a representative receptor molecule (PDB entry 1fg9, chain C [19]) is highlighted in Figures 2(a) and 2(b). All amino acid residues lining cavities in all four IFN*γ*R1 proteins complexed with IFN*γ* were combined, resulting in 52 residues used in subsequent* in silico* analysis.

### 3.2. *In Silico* Design of Variants

**Figure 3:**
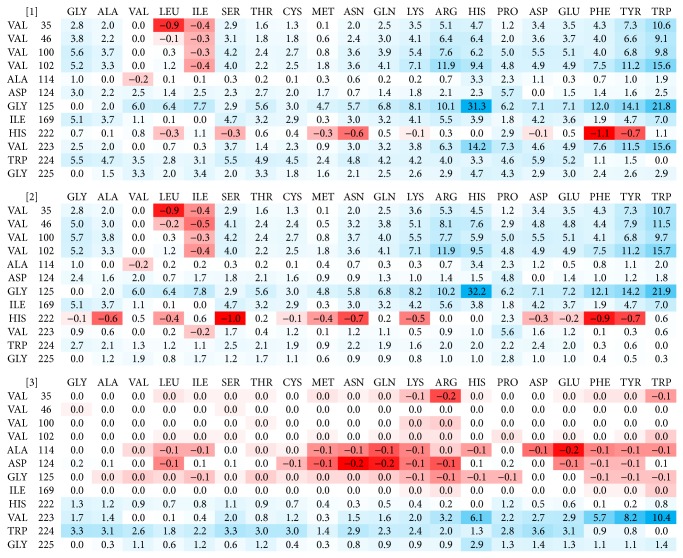
Color-coded values of free energy changes (ΔΔ*G*) of mutating the twelve cavity-lining residues of IFN*γ*R1. ΔΔ*G* values were calculated using the program FoldX for 500 MD snapshots and averaged. Red colored matrix fields indicate stabilization, blue ones destabilization. Shown are ΔΔ*G* values calculated for PDB 1fg9 [19]; receptor chain C. analogical matrices are calculated for 1fg9 receptor chain D, and for receptor chains B and E from the structure 1fyh [20]. (1) “ΔΔ*G* of folding of IFN*γ*R1 in complex” gauged the influence of mutations on the stability of the whole IFN*γ*/IFN*γ*R1 complex. (2) “ΔΔ*G* of folding of free IFN*γ*R1” estimated the effect of mutations on the stability of the isolated receptor. (3) “ΔΔ*G* of binding” of complex between IFN*γ*R1 and IFN*γ* made an estimate of change of the interaction between the receptor molecule and the rest of the complex.

All 52 amino acids lining the cavities of the receptor molecule were subject to the mutation analysis by FoldX. The resulting ΔΔ*G* values indicated potential for mutation leading to increasing the receptor affinity to IFN*γ*. The mutations were ordered by their ΔΔ*G* values and the first 50 best mutations from each crystal structure (200 mutations in total) were further analyzed. Of these 200 mutations, twelve positions were predicted in all four or at least three crystal structures. The twelve promising positions are highlighted in orange and yellow in Figure 2(c). Following the previous study [21], where we observed significant differences between ΔΔ*G* predicted directly from the crystal structures and from structures after molecular dynamics (MD) relaxation, we performed short (10 ns) MD simulations of the four crystal structures of complexes between wild type IFN*γ*R1 and IFN*γ*, and repeated the FoldX mutation analysis on 500 snapshots extracted from these MD trajectories. After averaging of the predicted ΔΔ*G* values for the twelve selected positions, we made the final selection of the four candidate mutations. The averaged ΔΔ*G* values resulting from these calculations for structure 1fg9, receptor chain C, are summarized in Figure 3. The final selection of the four variants is listed in Table 2 together with the changes of their binding free energies averaged over 500 MD snapshots from each of the four IFN*γ*/IFN*γ*R1 complexes in crystal structures 1fg9 and 1fyh.

**Figure 4:**
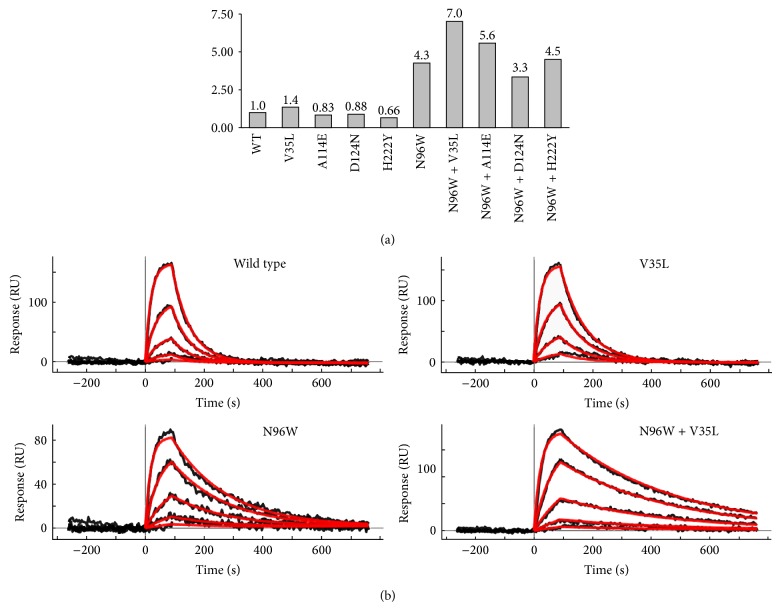
Affinities of the IFN*γ*R1 wild type (WT) and mutants to IFN*γ*SC obtained from SPR measurements. (a) Graph represents relative affinities of IFN*γ*R1 variants compared to WT. All selected “cavity” single amino acid mutation variants bind to the IFN*γ*SC with similar affinity as WT, but the V35L variant has slightly higher affinity itself and further increases the affinity of the “interface” mutant N96W if combined together. (b) SPR sensorgrams showing the interaction between IFN*γ*SC and selected IFN*γ*R1 variants. The V35L variant behaves similarly as WT displaying fast association and dissociation phases. Two variants (N96W and N96W + V35L) with higher affinities compared to WT bind IFN*γ*SC with slower dissociation phase, thus increasing the affinity. Measured SPR signal is in black and calculated fitted curves are in red; concentrations of IFN*γ*SC used for SPR measurements were as follows: 0.1, 0.3, 1.0, 3.0, and 9.0 nM.

Finally, the four consensus candidate mutations, which resulted as the best replacements of the WT sequence, were expressed, and characterized by SPR, CD, and thermal-based shift assay. The relative affinities of these four cavity-filling single mutants are shown in Figure 4(a) together with relative affinities of the double mutants combining the four cavity-filling mutations with mutation N96W.

As Table 2 and in detail Figure 3 show, the ΔΔ*G* calculations revealed only modest potential gains in interaction affinity, probably because of small cavity volumes as well as the fact that they are often lined by evolutionary highly conserved residues. As opposed to the interface mutations, where the predicted ΔΔ*G*s of IFN*γ*R1 stability and binding to IFN*γ* served as a sufficient criterion for the selection of affinity increasing mutations, there was no clear-cut rule for selecting internal cavity mutations that would result in improved interaction energy. We thus decided to test experimental consequences of combination of three types of ΔΔ*G* values calculated from the MD snapshots. To identify potentially favorable mutations, we combined ΔΔ*G* values of folding (ΔΔ*G* types (1) and (2) in the* in silico* protocol described in Materials and Methods) and of binding (type (3)). The first two mutations, V35L and H222Y, were predicted to increase ΔΔ*G* of folding to a similar extent for both the complexed and free IFN*γ*R1 (ΔΔ*G* (1) and (2)), while calculated values of their ΔΔ*G* of binding were virtually zero. The other two selected mutations, A114E and D124N, were predicted to slightly improve ΔΔ*G* of binding while both types of their ΔΔ*G* of folding were destabilizing. In the latter case, ΔΔ*G* of folding of free IFN*γ*R1 (type 2) was more unfavorable than ΔΔ*G* of folding of complexed IFN*γ*R1 (type 1). This means that the complex is predicted to be relatively more stable compared to the free IFN*γ*R1.

### 3.3. Experimental Determination of the Affinities between IFN*γ*R1 Variants and IFN*γ*SC

Computer-designed IFN*γ*R1 variants were expressed and purified and their affinities to IFN*γ*SC were determined by SPR measurements; relative affinities are plotted in Figure 4(a); SPR sensograms are depicted in Figure 4(b). The calculated *K*_*d*_ values showed that the four selected “cavity” single amino acid mutation variants bind to the IFN*γ*SC with similar affinity as WT; a modest increase was observed for the V35L variant. In line with our previous work, we decided to test to what extent the effect of two distant point mutations is additive. To this end, we combined the four cavity mutants designed here with the variant with the highest affinity designed previously, N96W. The results were quite encouraging: while affinity of one double mutant (N96W + H222Y) is neutral and one (N96W + D124) affinity actually decreased, two double mutants, N96W with A114E and V35L, had affinity increased compared to WT. The affinity increase of one of the double mutants, N96W + V35L, is significant, seven times higher than affinity of WT.

**Figure 5:**
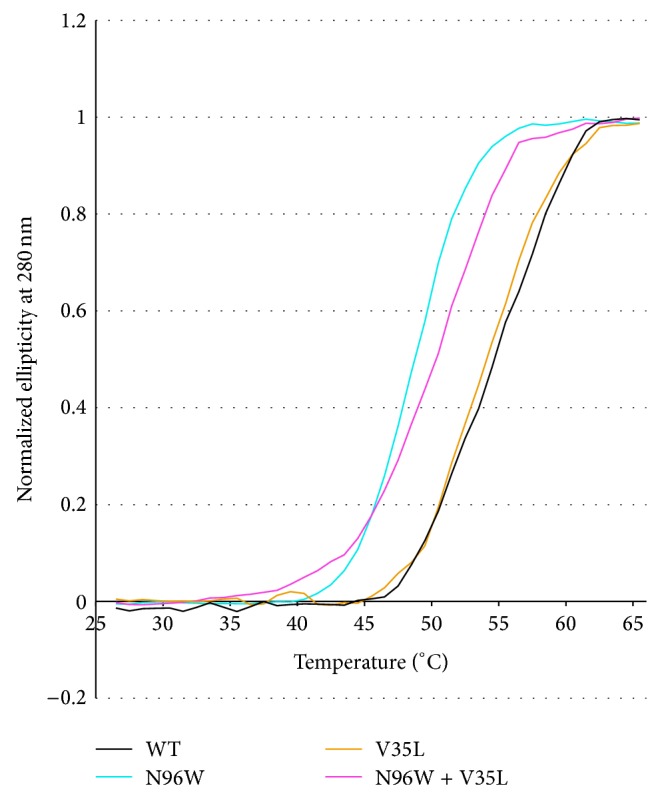
Normalized melting curves of IFN*γ*R1 variants measured by temperature-dependent near ultraviolet circular dichroism (CD) spectra. Each data point is from the intensity measured at 280 nm. IFN*γ*R1 WT, V35L, N96W, and N96W + V35L variants were measured in PBS buffer between 25 and 65°C at steps 1°C/minute. The melting temperature (*T*_*m*_) of IFN*γ*R1 variants was determined as 54°C for WT, 53°C for V35L, 50°C for N96W + V35L, and 48°C for N96W, respectively.

**Figure 6:**
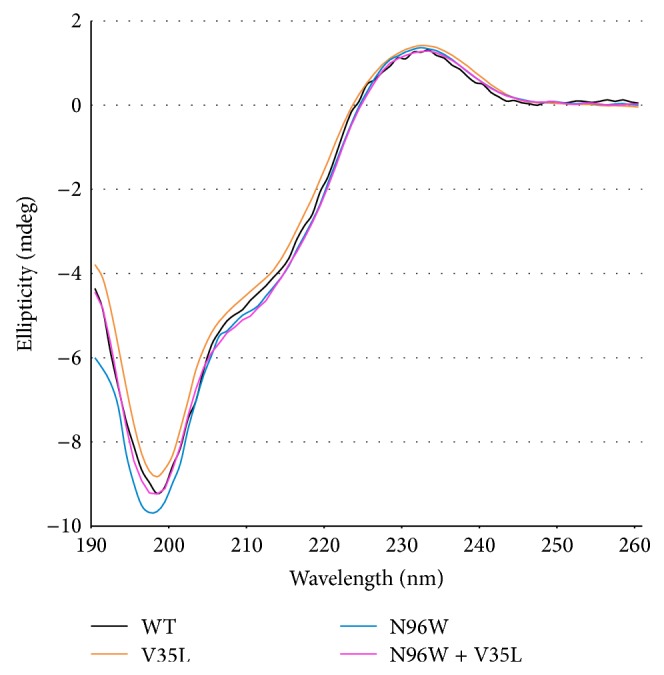
Circular dichroism (CD) spectra of IFN*γ*R1 variants (WT, N96W, V35L, and N96W + V35L) measured in water at 25°C. CD melting curves for the same variants are shown in Figure 5.

**Figure 7:**
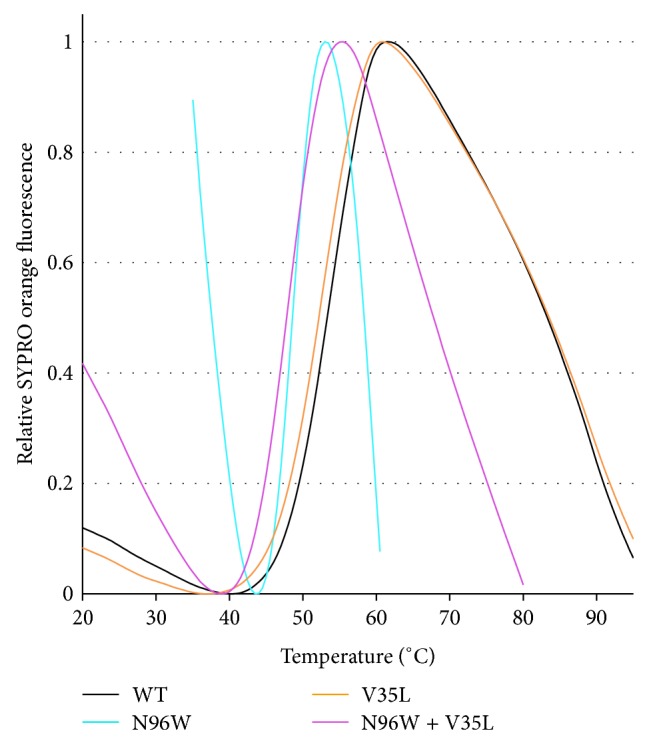
Melting temperatures of selected IFN*γ*R1 variants determined by thermal-based shift assay. Plotted are normalized data of reference-subtracted fluorescence intensities of IFN*γ*R1 WT, V35L, N96W, and N96W + V35L. The melting temperatures (*T*_*m*_) of IFN*γ*R1 variants were determined from the first derivatives of the curves plotted in the figure: 55°C for WT, 53°C for V35L, 49°C for N96W, and 48°C for N96W + V35L. The *T*_*m*_ values determined by temperature-dependent CD spectra and thermal-based shift assay are within 1°C the same.

The thermal stability (Figure 5) and secondary structure (Figure 6) of four IFN*γ*R1 variants, V35L, N96W, N96W + V35L, and WT, were studied by CD and their melting temperatures were confirmed by thermal-based shift assay (Figure 7); the CD-measured melting temperatures are 53, 48, 50, and 54°C, respectively. Both variants with the highest affinity, N96W and N96W + V35L, have melting temperatures lower than WT, so that mutation from asparagine to tryptophan at the position 96 apparently causes a decrease of IFN*γ*R1 thermal stability. However, the CD spectra of all four proteins are highly similar (Figure 6); their analysis provided virtually identical composition of the secondary structure elements dominated by the beta-sheet fractions indicating that no global structural rearrangements were caused by the mutations and the fold of these four variants is most likely the same. Moreover, the spectra are in agreement with the spectrum measured previously [35] for WT of IFN*γ*R1.

### 3.4. Analysis of Internal Dynamics of the IFN*γ*R1 Variants

**Figure 8:**
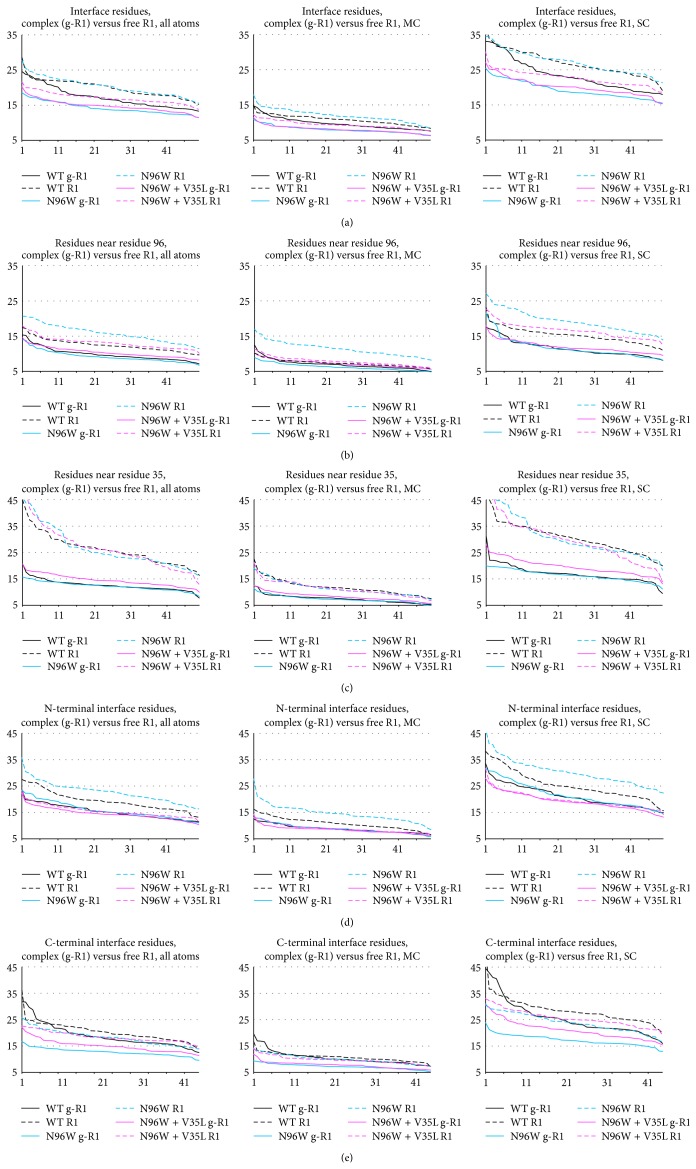
Ranked RMSF values collected at the last 50 ns of the 100 ns MD simulations of WT, N96W, and N96W + V35L variants of IFN*γ*R1. Solid lines labeled g-R1 denote RMSF values of the IFN*γ*/IFN*γ*R1 complex; dashed lines labeled R1 denote values of IFN*γ*R1 alone. The RMSF values are on the *y*-axis; the rank of the values (1–50) is on the *x*-axis. Shown are RMSF values of all atoms, main chain atoms (MC), and side chain atoms (SC) for the following residues: (a) all 40 interface residues (i.e., residue numbers 64, 65, 66, 67, 68, 69, 70, 71, 72, 73, 93, 95, 96, 97, 99, 115, 116, 118, 123, 164, 165, 166, 168, 170, 171, 186, 189, 190, 191, 192, 193, 197, 220, 221, 222, 223, 224, 225, 226, and 227); (b) residues within 6 Å of residue 96 (i.e., residue numbers 65, 66, 67, 91, 92, 93, 94, 95, 96, 97, 98, 119, 120, 121, and 224); (c) residues within 6 Å of residue 35 (i.e., residue numbers 32, 33, 34, 35, 36, 37, 46, 47, 48, 49, 100, 101, 102, 114, 115, 116, and 117); (d) the interface residues from the N-terminal domain (i.e., residues 64 to 123); (e) the interface residues from the C-terminal domain (i.e., residues 164 to 227).

To test how a cavity-filling mutation changes the flexibility of the receptor molecule in unbound and complexed states we analyzed root-mean square fluctuations (RMSF) of the selected variants. Comparison of RMSF sorted by their values, “ranked RMSF,” for WT, N96W, and N96W + V35L, are plotted in Figure 8 (solid lines for IFN*γ*/IFN*γ*R1 complexes, dashed lines for IFN*γ*R1 alone). These plots revealed significant differences between dynamics of the variants as is detailed below.

1. The interface residues of N96W and WT are more flexible in the free receptor than in the complex, while the flexibility of the interface residues of N96W + V35L is similar for the free and complexed receptor (Figures 8(a) and 8(d)). This indicates entropically more favorable binding of the N96W + V35L variant compared to the other two variants.
2. Interestingly, the origin of this behavior is different in the N-terminal and C-terminal domains of the IFN*γ*R1 molecule: in the N-terminal domain (Figure 8(d)), the flexibility of the interface residues of all variants is similar in the bound state, while being different in unbound state; they are most flexible in N96W and the least in N96W + V35L. In the C-terminal domain (Figure 8(e)), the flexibility of the three variants is similar in their free states, but it differs in the bound state between N96W, which has the lowest flexibility, and WT with the highest flexibility.
3. The V35L mutation stiffens the receptor nonlocally and makes especially the C-terminal interface residues more flexible in the bound state compared to the N96W mutant (Figure 8(e)).
4. To sum up, the V35L mutation brought flexibility of the free and complexed receptor closer together, indicating reduced entropy penalty of binding and resulting in the higher affinity of the N96W + V35L double mutant compared to N96W mutant.

Filling the cavity by hydrophobic groups as in the V35L mutation is stabilizing but not as much as would be implied by ΔΔ*G* of the removal of the corresponding hydrophobic group to water. A compensatory effect lowering a potential increase of the protein and/or complex stability has been observed previously [13] and a comparable decrease of stabilization was also predicted here by FoldX. Filling of a cavity may stabilize the interaction by several mechanisms, for example, by reducing the entropic penalty of complexation by stiffening interacting molecules in the free state, or indirectly by destabilization of the intermediate molten globule state rather than by stabilization of the folded protein [36]. These compensatory effects further illustrate complexity of protein-protein interactions (and/or folding) and the known limits of computational approaches to increasing protein-protein affinity [37].

An important issue potentially affecting reliability of FoldX predictions is the flexibility of the receptor molecule. The first round of FoldX ΔΔ*G* calculations based on the static crystal structures suggested one additional mutation, G225Y, as potentially increasing receptor affinity to IFN*γ*. Although further calculations using structures of snapshots from the MD simulations did not confirm this prediction, we expressed and characterized this mutation. The experimental data were in agreement with the MD-based prediction showing much lower binding affinity compared to the WT (the ratio of the respective *K*_*d*_ values was 0.4), and also the N96W + G225Y double mutant had a fairly low binding affinity (compared to WT, the ratio of the respective *K*_*d*_ values was 3.1, which is lower than for the N96W mutant). This observation can be explained by the structural properties of the receptor molecule. The loop region of IFN*γ*R1 containing the G225 residue is flexible and any residue at the position 225 is thus only a fraction of time in the geometry, in which it may increase the binding affinity. An important role of flexibility at the C-terminal part of the interacting IFN*γ* and IFN*γ*R1 is well illustrated by a study of IFN*γ* modified at its C-terminal side [38].

### 3.5. Sequence Conservation of Mutable Residues

We checked sequence conservation for the 12 positions selected by the FoldX calculations for potential cavity-filling mutations. Global alignment of 32 sequences of the extracellular part of IFN*γ*R1 from various organisms by Kalign as implemented in program Ugene [39] (Figure 2(c)) shows conservation between 40 and 98% for these positions; the position V35 is well conserved (80%). The independence of sequence conservation and its potential for stabilizing mutation filling-up protein cavity (“mutability”) contrasts with previously observed tight correlation between conservation and mutability for receptor residues interacting with IFN*γ* [21]: we tested several mutations of the interface residues S97 and E118, which were conserved at the 90% level (Figure 2(c)), namely, S97X (X = L, N, W) and E118X (X = M, F, Y, W), and they did not bind IFN*γ*SC at all (unpublished SPR data) despite the fact that binding of these mutants to IFN*γ* was predicted to be stronger than that of WT.

### 3.6. Relationship Between FoldX ΔΔ*G* Values and Naturally Occurring IFN*γ*R1 Variants

Interesting, albeit indirect, validation of the present FoldX predictions of ΔΔ*G* of mutations can be found among naturally occurring IFN*γ*R1 single-point mutations collected in the database of single nucleotide polymorphism (dbSNP) [40]. The database contains 25 nucleotide mutations at 22 unique positions of the extracellular part of the IFN*γ* receptor, which is studied here; these 22 positions are marked blue in Figure 2(c). Most of the ΔΔ*G* predictions for these natural mutants show neutral effect on the stability of free IFN*γ*R1 and on its complex with IFN*γ*. This is in agreement with the fact that only two of the natural mutants exhibit deleterious effects or are represented by a pathological phenotype.

## 4. Conclusions

We present a new computational strategy for designing higher affinity variants of a binding protein and show that it is possible to increase the affinity of a protein-protein interaction by mutations not at the interface, but in the interior cavities of a binding partner. The mutations were selected at positions lining internal cavities of one binding partner, and an* in silico* protocol identified mutations that would fill the protein cavities and increase the stability of the complex. We showed that the selection of such cavity mutations in interferon-*γ* receptor 1 (IFN*γ*R1) could be performed based on a combination of simple empirical force-field calculations and MD simulations. The mechanism by which the cavity mutations cause affinity increase is shown to be restriction of molecular fluctuations, which can be related to reduced entropy penalty upon binding [6, 7]. IFN*γ*R1 WT and all computationally designed receptor mutants were expressed, purified, and refolded, and the affinity towards the cognate protein, IFN*γ*SC, was measured by SPR. While single mutants showed roughly the same affinity as WT, double mutants combining cavity mutations with the best interface mutation obtained previously [21] were successful in further increasing the binding affinity.

The results demonstrate that mutating cavity residues is a viable strategy for designing protein variants with increased binding affinity. The comparison of computational data and experiments helped to further improve our understanding of forces governing protein-protein interactions. The newly obtained high-affinity binders of IFN*γ* could be developed into a new diagnostic tool. The significance of the present work can be seen in the fact that small ΔΔ*G* gains of cavity mutants led to significant increase of affinity when combined with more conventional mutations influencing the interface.

## Figures and Tables

**Table 1:** Cavities in the four molecules of the IFN*γ*R1 receptor in crystal structures 1fg9 [19] and 1fyh [20]. The receptor molecules are labeled by chain ID (chains C and D from 1fg9 and chains B and E from 1fyh).  Figure 2 shows cavities 1–8 as they project into the chain C of 1fg9.

	Surface [Å^2^]^*^	Number of residues lining the cavity^†^	Residues selected for mutation	Cavity observed in IFN*γ*R1 chain of	
				1fg9	1fyh
1	134	7	V35, A114	C D	—
2	133	5	—	—	B E
3	470	14	D124	C D	—
4	262	9	H222	C D	B E
5	120	6	—	C D	E
6	165	7	—	C D	E
7	177	7	—	D	B E
8	138	5	—	C	B

^∗^Surface calculated with a probe radius of 0.25 Å for cavities combined from all relevant receptor chains.

^†^Some residues are shared by neighboring cavities.

**Table 2:** Predicted changes of free energy changes (ΔΔ*G*) of the four selected IFN*γ*R1 variants with cavity-lining mutations relative to the wild type receptor. All energy values are in kcal/mol.

Variant	ΔΔ*G* of folding of IFN*γ*R1 in complex^*^	ΔΔ*G* of folding of free IFN*γ*R1^†^	ΔΔ*G* of binding of IFN*γ*R1/IFN*γ* complex^‡^	Sequence conservation^¶^
V35L	−0.88	−0.85	−0.02	80%
A114E	0.28	0.46	−0.20	60%
D124N	0.65	0.88	−0.21	40%
H222Y	−0.72	−0.69	0.15	40%

^∗^ΔΔ*G* of folding of IFN*γ*R1 bound to IFN*γ* measures the influence of mutations on the stability of the whole complex.

^†^ΔΔ*G* of folding of IFN*γ*R1 alone represents changes of the stability of the isolated receptor.

^‡^ΔΔ*G* of binding of the whole complex between IFN*γ*R1 and IFN*γ* estimates the change of the affinity between the receptor molecule and the rest of the complex.

^¶^Sequence conservation of amino acid residues at positions 35, 114, 124, and 222. It was based on the global alignment of 32 sequences of the extracellular part of IFN*γ*R1 (Figure 2(c)).
